# Obesity and its Association with Food Consumption, Diabetes Mellitus, and Acute Myocardial Infarction in the Elderly

**DOI:** 10.5935/abc.20160182

**Published:** 2016-12

**Authors:** Erika Aparecida da Silveira, Liana Lima Vieira, Thiago Veiga Jardim, Jacqueline Danesio de Souza

**Affiliations:** Universidade Federal de Goiás (UFG), Goiânia, GO - Brazil

**Keywords:** Obesity / complications, Aged, Food Consumption, Diabetes Mellitus, Diabetes Complications, Myocardial Infarction / complications, Risk Factors

## Abstract

**Background:**

Obesity affects a large part of elderly individuals worldwide and is considered a risk predictor for the development of chronic diseases such as cardiac diseases, the leading causes of death in the elderly population.

**Objective:**

To investigate the prevalence of obesity and associated factors, with emphasis on the occurrence of other diseases and on food consumption in elderly individuals treated at the Brazilian Unified Health System (Sistema Único de Saúde, SUS).

**Methods:**

Cross-sectional sampling study performed in the city of Goiânia (Brazil) including elderly individuals (≥ 60 years) receiving primary care. During home visits, we performed anthropometric measurements and applied a structured, standardized, and pre-tested questionnaire assessing socioeconomic, demographic and lifestyle conditions, occurrence of diseases, and food consumption. We performed multiple Poisson regression analysis using a hierarchical model and adopting a significance level of 5%.

**Results:**

We evaluated 418 elderly patients with a mean age of 70.7 ± 7 years. Their body mass indices had a mean value of 27.0 kg/m^2^ and were higher in women than in men (27.4 kg/m^2^ versus 26.1 kg/m^2^, respectively, p = 0.017). Obesity had a prevalence of 49.0%, a risk 1.87 times higher between the ages of 60-69 years and 70-79 years, and a rate 1.4 times higher among individuals with more than four morbidities. On multivariate analysis, the factors associated with obesity were age 60-69 and 70-79 years, inadequate consumption of whole-wheat grains and adequate consumption of fruit, musculoskeletal diseases, diabetes mellitus, and acute myocardial infarction.

**Conclusions:**

Obesity had a high prevalence in the evaluated elderly population and was associated with food consumption, musculoskeletal disease, diabetes mellitus, and acute myocardial infarction.

## Introduction

Population aging is a complex, multifactorial, and global phenomenon occurring at an accelerated pace. It is estimated that in 2025, Brazil will have the sixth largest elderly population in the world, with 32 million individuals, which will represent 13% of the total population in the country.^1^

The process of aging is a reality accompanied by changes in the population's health profile, with emphasis on the occurrence of chronic non-communicable diseases (CNCDs), resulting in greater demand for health services and associated with loss of autonomy, decreased quality of life, and increased mortality among elderly individuals.^1,2^

Among all CNCDs, cardiovascular diseases (CVDs) should be highlighted. These are the main responsible for morbidity and mortality in Brazil and worldwide, and affect in particular the elderly population.^3-5^

In parallel with demographic and epidemiological changes, the nutritional scenario has seen a decline in malnutrition and a significant increase in the prevalence of obesity.^6,7^ Obesity is not simply an increase in weight, but an excess of body fat. Aging is associated with an increase in body fat and changes in fat distribution pattern, with an increase of 20% to 30% in total body fat (2-5% per decade after the age of 40 years).^8,9^ Obesity is considered a risk factor for other CNCDs including diabetes mellitus (DM), hypertension, dyslipidemia, cancer, and CVDs.^10^

The prevalence of obesity in non-institutionalized elderly individuals using the Brazilian Unified Health System (SUS) remains unknown. In addition, studies on this subject have not provided information about the factors associated with obesity, or the association of obesity with dietary consumption variables and the occurrence of other diseases, which restricts the understanding of this condition. Given this context, this study aimed to estimate the prevalence of obesity and verify its association with sociodemographic factors, lifestyle with an emphasis on food consumption, and presence of comorbidities in community-dwelling elderly individuals receiving primary care at the SUS.

## Methods

Cross-sectional study inserted in the *Projeto Idoso Goiânia* (Project Elderly Goiânia),^11-14^ which assessed the health and nutritional conditions in community-dwelling elderly individuals receiving care at the SUS in Goiânia (GO). We included non-institutionalized elderly individuals aged 60 years or older, of both sexes, living in Goiânia, treated at the SUS, and with appointments carried out within 12 months prior to the survey. We excluded from the sample bedridden elderly individuals or those with other impediments for anthropometric measurements or inability to answer the questionnaire.

We conducted a multistage sampling procedure considering the elderly population living in each of the nine Health Districts (HD) of the municipality. The calculation of the sample size was conducted *a posteriori*, with the objective of verifying whether the sample size of the matrix project would fit this study.^11-14^ For that, we considered obesity as an outcome, the female sex as an exposure variable, and the following parameters: 95% confidence interval (95%CI), 80% power, ratio of not exposed:exposed of 1:1.9, prevalence ratio (PR) of 1.9, and prevalences of the disease among non-exposed (men) of 16.9% and among exposed (women) of 32.11%, according to information derived from the database. The sample size was estimated at 310 elderly, and to stratify and control for confounding factors, we added 10%, totaling a final sample of 341 elderly individuals. Thus, the sample size of the matrix project (418 elderly) was sufficient to ensure the validity and the statistical power of the present study.

We performed home visits between November 2008 and March 2009, in which we administered a structured and standardized questionnaire that was pre-tested in a pilot study. Anthropometric measurements, such as weight and height, were performed after standardization, according to the procedures recommended by Lohman et al.^15^ Weight was measured in kilograms, using a portable digital electronic scale (Tanita) properly calibrated, with a capacity of up to 150 kg and accurate to 100 g. Height was measured with a 2-meter tape measure accurate to 0.1 cm and fixed to a flat wall without a baseboard, with the help of a plumb line and a wooden set-square. Weight and height were measured in duplicate, and the arithmetic mean of the measurements was used as the result. Body mass index (BMI) was defined according to the criteria proposed by Lipschitz.^16^ The nutritional status was defined by the BMI, as per the criteria discussed and recommended by Silveira et al.:^17^ BMI < 22 kg/m^2^ as low weight, 22 to 27 kg/m^2^ as eutrophic, and BMI > 27 kg/m^2^ (the outcome variable) as obesity. This classification considers the body composition changes that are characteristic of aging, taking into consideration that this cut-off point has a greater agreement with the body fat percentage and relates best to various complications in the elderly.^8,17^

The measurement of the systolic and diastolic blood pressure (SBP and DBP, respectively) was obtained from the mean of two measurements with a semiautomatic device (Omron HEM-705CP) according to the criteria established by the VI Brazilian Hypertension Guidelines.^18^ Elderly patients with a SBP ≥ 140 mmHg and/or a DBP ≥ 90 mmHg or receiving pharmacological treatment for hypertension were considered as being hypertensive.^18^

The independent variables analyzed were: socioeconomic and demographic factors (gender, age, skin color, life with a partner, years of study, economic stratum, and parity); lifestyle (smoking, alcohol consumption, and sedentary lifestyle); food consumption (fruits, legumes, vegetables, whole-wheat cereal, fat-free milk, processed meats, packaged snack foods, sweets, and sodas), and occurrence of morbidities (number of morbidities, DM, hypothyroidism, musculoskeletal diseases, acute myocardial infarction [AMI], stroke, and hypertension).

The economic stratum was determined according to the economic classification criteria of the *Associação Brasileira de Empresas de Pesquisa* (Brazilian Association of Researching Companies - ABEP, 2008).^19^ In relation to the consumption of alcoholic beverages, we verified the type of beverage, the frequency and amount consumed during the previous week (in doses, bottles, cups, or glasses) to then determine the daily amount of ethanol (in grams) consumed by the elderly individuals. We considered as a cardiovascular risk factor the consumption of alcoholic beverages exceeding 30 g/day for men and 15 g/day for women or individuals with low weight.^18^

The presence of morbidities was analyzed and categorized according to the International Statistical Classification of Diseases and Related Health Problems - ICD10 (2008).^20^ The variable sedentary lifestyle was assessed, categorized, and classified according to the presence of all the following categories: inactive during leisure time (no physical activity during leisure); inactive in household chores (absence of heavy domestic activity of at least 3 days a week with a total duration of 3 hours); inactive during work (self-reported), such as seating most of the time or only performing activities with little physical effort, and inactive during commute to work (commute by car, motorcycle, bus, or less than 10 minutes of walking or biking).^21^

To measure food consumption, we used the Frequency of Food Consumption Questionnaire (FFCQ), a qualitative tool that includes a list of foods and frequency of consumption (daily, weekly, monthly, rarely, or never). In the data analysis, the variables were categorized as having adequate or inadequate consumption, according to the recommendation of the Dietary Guidelines for the Brazilian Population, after adjustments based on the frequency of consumption of each food (daily, weekly) and not on the number of servings, considering that the FFCQ does not provide information on portions: fruits (adequate - daily consumption), legumes and vegetables (adequate - daily consumption), fat-free milk (adequate - daily consumption), whole-wheat cereal (adequate - daily consumption); fats (adequate - consumption of less than two items daily including margarine, butter, mayonnaise, lard/pork rinds/pork cracklings, bacon, greasy meats, and fried foods); hamburger/processed meat/ packaged snack foods (adequate - consumption of at least one item reported as rare or never); sweets and sodas (adequate - consumption reported as not occurring daily); pies, pastries, cakes, cookies, or delicacies (adequate - consumption fewer than three times per week).^7^

The statistical analysis was performed using the program Stata 8.0 and the database was entered twice in EpiData 3.1. We performed a descriptive analysis of obesity, the variable of interest, and conducted a simple Poisson regression with 95%CI to assess its association with the exposure variables.

On multivariate analysis using a hierarchical model with Poisson regression, we included in the bivariate analysis those variables that showed significance below or equal to 0.20. In the proposed hierarchical model, the socioeconomic and demographic variables comprised the most distal level (level 1) and the lifestyle and health conditions variables comprised the most proximal level (level 2). The variables included in the model were age, color, smoking, fruits, vegetables and legumes, whole-wheat cereal, fat-free milk, number of morbidities, DM, hypothyroidism, musculoskeletal diseases, AMI, stroke, and hypertension.

We initiated a hierarchical analysis model using the variables from the most distal level (level 1). The variables with a p value above 0.05 were removed from the multivariate model.

We should emphasize that the participation of the elderly individuals was voluntary and happened after they signed an informed consent form approved by the institution's Research Ethics Committee, according to Resolution No. 466/2012 of the National Health Council.

## Results

We evaluated 418 elderly patients with a mean age of 70.7 ± 7 years. The group's BMI had a mean value of 27.0 kg/m^2^ and was higher in women than men (27.4 kg/m^2^ and 26.1 kg/m^2^, respectively, p = 0.017). All continuous variables had a normal distribution.

Obesity had an overall prevalence of 49.0% (95%CI 44.2 -54.0%), including 51.1% (95%CI 45.0- 57.1%) in women and 45.1% (95%CI 36.7-53.6%) in men, without statistically significant difference between them.

Obesity was associated with age (p = 0.034) in the age ranges of 60-69 and 70-79 years. The risk of an individual being obese in these two age groups was 1.87 times greater than among those in the group with 80 years or more (Table 1).

**Table 1:** Distribution of the sample, prevalence of obesity in the elderly, and association according to socioeconomic and demographic variables. Projeto Idosos Goiânia (Project Elderly Goiânia), Brazil (n = 418)

Variables	Sample distribution n (%)	Prevalence n (%)	PR (95%CI)	p value*
**Sex**				0.254
Female	276 ( 66.03)	141 ( 51.09)	1.13 (0.91-1.40)	
Male	142 ( 33.97)	64 (45.07)	1.00	
**Age**				0.034
60 to 69	203 (48.56)	105 (51.72)	1.87 (1.15-3.02)	
70 to 79	168 (40.19)	87 (51.79)	1.87 (1.15-3.04)	
80 or more	47 (11.24)	13 (27.66)	1.00	
**Skin color**				0.178
White	194 (46.41)	102 (52.58)	1.14 (0.94-1.39)	
Brown and Black	224 (53.59)	103 (45.98)	1.00	
**Life with a partner**				0.267
Yes	229 (54.78)	118 (51.53)	1.12 (0.92-1.37)	
No	189 (45.22)	87 (46.03)	1.00	
**Years of study^‡^**				0.852
Illiterate	112 (29.95)	52 (46.43)	1.00	
1 to 4	154 (41.18)	79 (51.30)	1.10 (0.86-1.42)	
5 to 8	72 (19.25)	35 (48.61)	1.05 (0.77-1.43)	
9 or more	36 (9.63)	19 (52.78)	1.14 (0.79-1.64)	
**Economic stratum**				0.854
A/B	63 (15.29)	30 (47.62)	1.00 (0.74-1.36)	
C	193 (46.84)	97 (50.26)	1.06 (0.85-1.32)	
D/E	156 (37.86)	75 (47.44)	1.00	
**Parity^¦^**				0.512
zero	21 (7.66)	9 (42.86)	1.00	
1 to 3	55 (20.07)	27 (49.09)	1.14 (0.65-2.01)	
4 to 6	83 (30.29)	48 (57.83)	1.35 (0.79-2.29)	
7 or more	115 (41.97)	57 (49.57)	1.16 (0.68-1.96)	

* Wald test,

‡ missing data for 44 individuals,

¦ data related to 274 women, PR: prevalence ratio; 95%CI: 95% confidence interval.

As for variables related to dietary consumption, we observed in the bivariate analysis that obesity was associated with consumption of fruits, legumes, whole-wheat cereals, and fat-free milk (Table 2).

**Table 2:** Distribution of the sample, prevalence of obesity in the elderly, and association according to the variables lifestyle and dietary intake. Projeto Idosos Goiânia (Project Elderly Goiânia), Brazil (n = 418)

Variables	Sample distribution n (%)	Prevalence n (%)	PR (95%CI)	p value*
**Smoking**				0.120
Non-smokers	198 (47.37)	100 (50.51)	1.64 (1.00-2.68)	
Ex-smoker	181 (43.30)	93 (51.38)	1.67 (1.02-2.73)	
Smoker	39 (9.33)	12 (30.77)	1.00	
**Consumption of alcoholic beverages**				0.259
No	354 (84.69)	178 (50.28)	1.19 (0.88-1.62)	
Yes	64 (15.31)	27 (42.19)	1.00	
**Sedentary lifestyle**				0.531
Yes	230 (55.02)	116 (50.43)	1.06 (0.87-1.30)	
No	188 (44.98)	89 (47.34)	1.00	
**Fruits**				0.011
Inadequate	233 (56.01)	102 (43.78)	1.00	
Adequate	183 (43.99)	103 (56.28)	1.28 (1.06-1.56)	
**Legumes and vegetables**				0.035
Inadequate	316 (75.60)	147 (46.52)	1.00	
Adequate	100 (24.04)	58 (58.00)	1.24 (1.02-1.53)	
**Legumes**				0.022
Inadequate	73 (17.51)	44 (60.27)	1.29 (1.04-1.60)	
Adequate	344 (82.49)	161 (46.80)	1.00	
**Whole-grain cereal**				0.031
Inadequate	367 (88.22)	189 (51.50)	1.58 (1.04-2.39)	
Adequate	49 (11.78)	16 (32.65)	1.00	
**Fat-free milk**				0.047
Inadequate	357 (85.61)	169 (47.34)	1.00	
Adequate	60 (14.39)	36 (60.00)	1.27 (1.00-1.60)	
**Fats**				0.842
Inadequate	44 (10.55)	21 (47.73)	1.00	
Adequate	373 (89.45)	184 (49.33)	1.03 (0.74-1.43)	
**Hamburger/processed meats/packaged snack foods**				0.449
Inadequate	173 (41.59)	81 (46.82)	1.00	
Adequate	243 (58.41)	123 (50.62)	1.08 (0.88-1.32)	
**Sweets**				0.596
Inadequate	24 (5.76)	13 (54.17)	1.11 (0.76-1.62)	
Adequate	393 (94.24)	192 (48.85)	1.00	
**Sodas**				0.896
Inadequate	23 (5.52)	11 (47.83)	1.00	
Adequate	394 (94.98)	194 (49.24)	1.03 (0.66-1.59)	

* Wald test, PR: prevalence ratio; 95%CI: 95% confidence interval.

We found that 73.4% of the elderly individuals had two or more morbidities, and that the probability of developing obesity was 1.4 times greater among those with four or more morbidities (p = 0.023). We observed a higher prevalence of obesity in elderly individuals with AMI, hypothyroidism, DM, and musculoskeletal diseases; only hypothyroidism was not significantly associated with obesity in the bivariate analysis (Table 3).

**Table 3:** Distribution of the sample, prevalence of obesity in the elderly, and association according to health conditions. Projeto Idosos Goiânia (Project Elderly Goiânia), Brazil (n = 418)

Variables	Sample distribution n (%)	Prevalencen (%)	PR (95%CI)	p value *
**Nº of morbidities**				0.023
0 to 1	111 (26.56)	47 (42.34)	1.00	
2 to 3	214 (51.20)	102 (47.66)	1.12 (0.87-1.46)	
4 or more	93 (22.25)	56 (60.22)	1.42 (1.08-1.87)	
**Diabetes**				0.001
No	319 (76.50)	144 (45.14)	1.00	
Yes	98 (23.50)	61 (62.24)	1.38 (1.33-1.68)	
**Hypothyroidism**				0.102
No	396 (94.74)	191 (48.23)	1.00	
Yes	22 (5.26)	14 (63.64)	1.32 (0.96-1.84)	
**Musculoskeletal d.**				0.036
No	277 (66.27)	126 (45.49)	1.00	
Yes	141 (33.73)	79 (56.03)	1.23 (1.01-1.50)	
**Gastrointestinal d.**				0.992
No	369 (88.28)	181 (49.05)	1.00 (0.74-1.36)	
Yes	49 (11.72)	24 (48.98)	1.00	
**Respiratory d.**				0.234
No	373 (89.23)	187 (50.13)	1.25 (0.86-1.82)	
Yes	45 (10.77)	18 (40.00)	1.00	
**Infectious d.**				0.922
No	394 (94.26)	193 (48.98)	1.00	
Yes	24 (5.74)	12 (50.00)	1.02 (0.67-1.54)	
**Neoplastic d.**				0.816
No	407 (97.37)	200 (49.14)	1.08 (0.56-2.08)	
Yes	11 (2.63)	5 (45.45)	1.00	
**Hypertension**				0.149
No	82 (19.62)	34 (41.46)	1.00	
Yes	336 (80.38)	171 (50.89)	1.23 (0.93-1.62)	
**Stroke**				0.076
No	402 (96.4)	202 (50.25)	2.51 (0.91-6.95)	
Yes	15 (3.60)	3 (20.00)	1.00	
**Acute myocardial infarction**				0.011
No	408 (97.84)	198 (48.53)	1.00	
Yes	9 (2.16)	7 (77.78)	1.60 (1.11-2.30)	

* Wald test, d: diseases according to the International Statistical Classification of Diseases and Related Health Problems - ICD10, PR: prevalence ratio; 95%CI: 95% confidence interval.

On multivariate analysis, the factors associated with obesity were: age group, with equal risk in the age ranges from 60-69 years (PR = 1.87, 95%CI 1.15-3.02) and 70-79 years (PR = 1.87, 95%CI 1.15-3.04); adequate consumption of fruits and inadequate consumption of whole-wheat cereals; and occurrence of DM, musculoskeletal diseases, and AMI (Table 4).

**Table 4:** Final model of multivariate analysis of factors associated with obesity in the elderly. Projeto Idosos Goiânia (Project Elderly Goiânia), Brazil

Variables	Adjusted PR	95%CI	p value*
**1st Level**			
**Age**			
60 a 69	1.87	1.16-3.03	0.011
70 a 79	1.87	1.15-3.04	0.011
80 or more	1.00		
**2nd Level**			
**Fruits**			
Inadequate	1.00		
Adequate	1.28	1.06-1.55	0.011
**Whole-grain cereal**			
Inadequate	1.70	1.14-2.52	0.009
Adequate	1.00		
**Diabetes**			
No	1.00		
Yes	1.40	1.16-1.70	0.001
**Musculoskeletal diseases**			
No	1.00		
Yes	1.25	1.03-1.51	0.024
**Acute myocardial infarction**			
No	1.00		
Yes	1.60	1.07-2.39	0.022

* Wald test, PR: prevalence ratio; 95%CI: 95% confidence interval.

## Discussion

Population aging is a global reality, and the understanding of different variables associated with this process is crucial. In this study, obesity and related factors were investigated in a group of elderly individuals receiving care at the SUS in a capital city in the midwest region of Brazil.

The socioeconomic variables of the studied population are similar to those found in epidemiological data of the Brazilian urban population published by IBGE.^1^ Such similarities are more evident when comparisons are made with the information relating to areas closer to the central region of the country, mainly in the variables economic status and schooling (years of study). It is worth noting that the official information available^1^ includes individuals above the age of 60 years in the same category, without age subdivisions as in this study.

The prevalence of obesity among the elderly was extremely high (49%) and different from that obtained in the Vigitel,^22^ a large Brazilian population study in which the prevalence of obesity in the elderly population was 30%. Even in the United States, where obesity is a serious public health problem, the prevalence of obesity in another large population study was less than 35%.^23^ This difference may be explained by the fact that many population-based data on the prevalence of obesity use self-reported weight and height, which are subject to errors, especially in the elderly population. In contrast, the evaluation of height and weight for BMI calculation in the present study was obtained from all elderly individuals.

We observed a high prevalence of obesity in elderly individuals aged 60-79 years and a decrease after the age of 80 years, with a significant association of obesity with age range. Different studies have found this association, which may be due to a survival bias, *i.e.*, a lower life expectancy among obese individuals, who are unlikely to reach the age of 80 years.^17,24^

The mean BMI among the evaluated women was statistically superior to that among men, which is in line with other studies that established this association.^2,17,24^ A possible explanation for this fact is the greater tendency of accumulation of visceral fat after menopause, in addition to an increased life expectancy in women.

Aging occurs concomitantly with the onset of CNCDs and increase in comorbidity risk.^4,17,23^ Among the elderly analyzed in the present study, 73.4% reported having at least two diseases. Although the number of morbidities was not associated with obesity on multivariate analysis, it is important to highlight that the prevalence of obesity increased in parallel with the increase in the number of diseases, reaching 60.2% in those with four or more morbidities. Another study with elderly individuals found a significantly greater mean number of morbidities in elderly obese when compared with eutrophic and low-weight individuals.^11^

The prevalence of obesity was significantly greater in elderly patients with a previous diagnosis of DM and AMI, a finding that has been observed in other studies addressing this issue.^16,25^

Obesity, particularly visceral and of long duration, is a fundamental part in the pathogenesis of type 2 DM. Although the evaluation of the type of obesity was not the focus of this study, the advanced age suggests a prolonged exposure to obesity, justifying the association encountered. National data clearly reflect a higher prevalence of DM at more advanced ages.^26^ Another aspect to be highlighted is that DM treatment alone can contribute to weight gain, corroborating the data observed.^27^

The association between obesity and AMI is well established;^28^ however, some particularities in the elderly population deserve discussion. In some specific conditions, such as non-ST-elevation AMI, the mortality of elderly obese patients is lower.^28^ This finding has been observed in some studies^29-31^ assessing mortality due to other diseases related to obesity and overweight and is known as the obesity paradox. We did not evaluate or compare mortality rates in the present study, but we demonstrated a clear association of obesity with AMI.

The presence of these pathologies associated with obesity deserves health interventions with an emphasis on weight loss in order to also assist in the control of DM and prevent new cardiovascular events that increase the risk of death or sequelae. It should be mentioned, as a control measure, the regular practice of physical activity, which may delay the emergence of CNCDs. Of note, moderate exercise practice is responsible for reducing the risk of death by 20 to 25% in individuals with cardiac disease.^2,6,32^

Obesity was also associated with musculoskeletal diseases, confirming the higher risk of development of these diseases among obese individuals, often by excessive load, especially on the knee and hip. This relationship suggests the establishment of a greater dependence in activities of daily living, in addition to a greater fragility of elderly individuals.^33,34^

A low daily consumption of fruits, vegetables and legumes, whole-wheat cereals, and fat-free milk was characteristic in this study population. The nutritional transition is marked by excessive consumption of sugars, fats, and sodas, and insufficient consumption of fruits, vegetables, and fiber, pointing toward unfavorable trends in dietary patterns.^35,36^ The refinement of the grain affects the nutritional quality of the food, which becomes lower in vitamins and minerals, in addition to dramatically reducing the dietary fiber content, which affects the glycemic index of foods and acts to control weight.^36,37^ The low daily intake of fruits, vegetables, cereals, and milk and dairy products, and high consumption of sugar, accompanied by inadequate intake of essential micronutrients by elderly individuals has been demonstrated in different studies.^36-40^

The prevalence of obesity was significantly higher in elderly with adequate intake of fruits, while for whole-wheat grains, the risk of obesity was higher in individuals with inadequate intake of such food. This is a very interesting result that can be understood within the characteristics of the elderly sample. Since these elderly individuals had received primary care in the prior year and had diagnosed chronic diseases such as DM and obesity, they had probably already received interventions from the health care team regarding healthy eating. The association with fruit consumption may be related to a habit of more easily incorporation into the diet than the consumption of whole grains.

It should be remembered that the assessment of food intake has some limitations; however, this is inherent to the methodology of dietary surveys, since there is no gold-standard method for that. Another aspect refers to the possibility of recall bias, but this is another intrinsic limitation of indirect methods of assessment of food intake in the elderly.

A limitation that deserves to be highlighted is related to the fact that the sample comprised elderly individuals from a midwestern city capital, thus, representative of a population living in an urban area. Therefore, it is not necessarily representative of the entire population of the state, or even from a country with continental dimensions and full of regional inequalities, such as Brazil.

Given the findings, it should be highlighted the importance of monitoring the nutritional status of elderly individuals and creating actions of primary health intervention aimed at the control of obesity and prevention of cardiovascular events, in order to prevent the aggravations and consequences associated with the problem, help promote health, and improve the quality of life of elderly individuals treated at the SUS in Brazil.

## Conclusions

In the evaluated population of elderly individuals, we observed a high prevalence of obesity, which was associated with food intake, musculoskeletal disease, DM, and AMI.
